# A case of subdural block triggered posterior reversible encephalopathy syndrome: A case report

**DOI:** 10.1097/MD.0000000000043519

**Published:** 2025-07-25

**Authors:** Haojie He, Jun He, JianKe Kuai

**Affiliations:** a Department of Anesthesiology, Xi’an No. 3 Hospital, The Affiliated Hospital of Northwest University, Xi’an, Shaanxi, PR China.

**Keywords:** anesthesia, caesarean section, case report, postdural puncture headache (PDPH), posterior reversible encephalopathy syndrome (PRES), subdural block

## Abstract

**Rationale:**

Posterior reversible encephalopathy syndrome (PRES) is a relatively rare neurological disorder in obstetrics. Clinically, the most common precipitating factors are hypertension, immunosuppressive agents, or chemotherapy drugs. It is extremely rare for PRES to be induced by the entry of local anesthetic into the subdural space.

**Patient concerns:**

In this case report, we present an instance where a patient experienced blurred vision, and even blindness, following an accidental subdural block administered by the anesthetist, and was subsequently diagnosed with PRES. The patient was a 30-year-old parturient who developed blurred vision and headache after surgery, which further progressed to blindness.

**Diagnoses:**

Based on the clinical manifestations of headache, blurred vision, and even blindness in the patient, in conjunction with the imaging findings of abnormal signals in the bilateral temporal, parietal, and occipital lobes of the brain as well as the cerebellar hemispheres, a diagnosis of PRES was made.

**Interventions:**

Fluid restriction, diuresis, antihypertensive treatment, and neurotrophic support.

**Outcomes:**

The patient’s headache symptoms were rapidly alleviated, and her vision gradually returned to normal, leading to a successful recovery and discharge from the hospital.

**Lessons:**

This is an extremely unusual case. The accidental subdural block affected the vasoconstriction of the cerebral blood vessels, which induced PRES. Given that postoperative headaches in obstetric patients are often attributed to postdural puncture headache, the initial misdiagnosis and mistreatment by the obstetrician nearly led to a severely adverse prognosis for the patient.

## 1. Introduction

Subdural block was proposed by scholars quite early on,^[1]^ yet it is seldom encountered in clinical practice, often only diagnosed through retrospective analysis, and our understanding of it remains incomplete. Posterior reversible encephalopathy syndrome (PRES) is a relatively common condition in neurology, with current beliefs that the triggers include hypertension, kidney disease, preeclampsia and eclampsia, immunosuppressants, autoimmune diseases, and so on.^[2–4]^ The 2 diseases seemingly have no connection, but in this clinical case, we observed that a subdural block may have triggered PRES.

## 2. Case report

A healthy 30-year-old woman in childbirth was subjected to a cesarean section due to the large size of the fetus and cephalopelvic disproportion. Preoperative laboratory tests were within normal limits, and her blood pressure was also within the normal range. The admission diagnosis was a first pregnancy with no previous deliveries, at 40 weeks and 6 days of gestation; the pregnancy was complicated by hypothyroidism and allergic rhinitis.

The anesthetist chose the L2-3 interspace for 18-gauge epidural needle insertion. Upon loss of resistance, a negative pressure test was performed using normal saline to confirm the epidural space. Subsequently, a 27-gauge spinal needle was inserted. However, the needle reached the bony structure but failed to enter the subarachnoid space. After repositioning the epidural needle and reinserting the spinal needle, cerebrospinal fluid (CSF) could still not be aspirated. The patient reported an electric shock sensation in her lower limbs. Therefore, the subarachnoid block was abandoned. Instead, an 20-gauge epidural catheter was then inserted to a depth of 4 cm (epidural space), with no return flow of blood or CSF. The catheter was secured, and a test dose of 2% lidocaine 3 mL was administered. After an uneventful 8-minute observation period, the initial dose of 2% lidocaine 4 mL plus 1% ropivacaine 4 mL was given (the local anesthetic was preservative-free). Within just 6 minutes, the patient exhibited irritability, mild respiratory distress, and discomfort in the lumbar and thoracic regions. Her blood pressure was recorded at 121/73 mm Hg, with an oxygen saturation of 97%. The anesthetist considered at this point that the high level of the anesthetic block might have caused the respiratory distress, so he administered oxygen via a face mask and closely monitored the vital signs. The anesthetic block was effectively established for the surgery, with motor function in the lower limbs preserved. Propofol 80 mg was given intravenously for sedation to ease the patient’s anxiety, and the surgery proceeded without incident. The procedure took 40 minutes, and a healthy fetus was successfully delivered. No further local anesthetics were administered via the epidural catheter during the surgery. Prior to leaving the operating room, the parturient reported mild lip numbness, sensory loss below the neck, and numbness in both upper limbs with a muscle strength of grade 4, yet motor function in all limbs was maintained and vital signs remained stable. The anesthetist was highly experienced. The dose of medication administered via the epidural route was not excessive, and the rate of drug administration was typical for routine epidural anesthesia. However, the clinical presentation was that of a high-level block. Upon assessing the block level, it was found that the sensory levels on both sides were not entirely symmetrical (propofol had masked this phenomenon, which was not detected in a timely manner during surgery), and there was incomplete motor block. At this point, the anesthetist considered the possibility of an epidural subdural block. Nevertheless, the parturient’s vital signs were stable, and she was not experiencing any respiratory distress. Therefore, she was returned to the ward for further monitoring. Four hours later, the parturient’s symptoms had entirely resolved, and muscle strength in all limbs had returned to normal.

**Figure 1.**
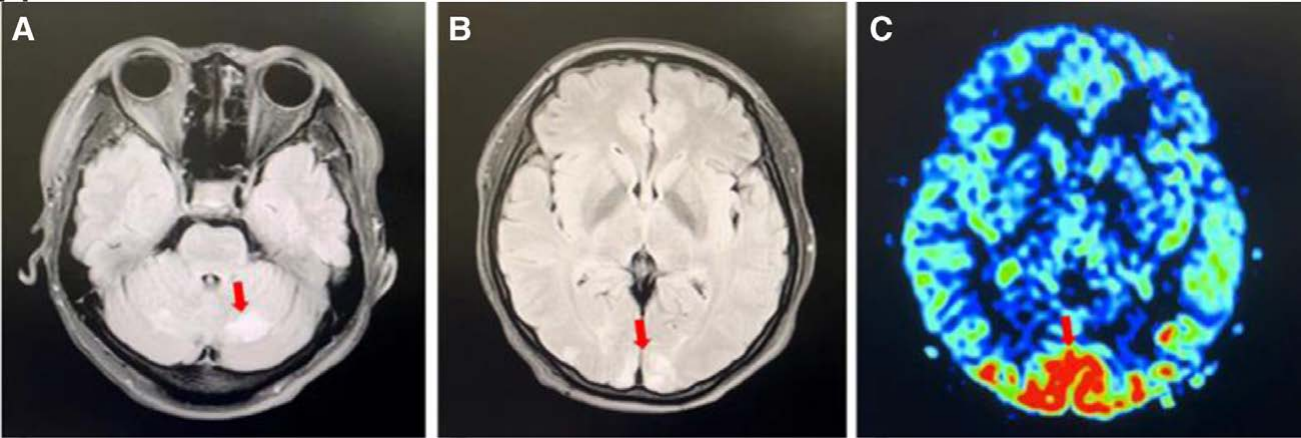
Brain MRI and cerebral blood perfusion images of the patient at the time of diagnosis of PRES. (A) Abnormal signal intensity in the posterior lobe of the cerebellum on MRI scan. (B) Abnormal signal intensity in the occipital lobe on MRI scan. (C) There is abnormal blood perfusion in the occipital lobe on functional MRI. MRI = magnetic resonance imaging, PRES = posterior reversible encephalopathy syndrome.

On the second postoperative day, the parturient complained of pain in the lumbar and spinal region, limited movement of the head and neck, and headache. A cranial magnetic resonance imaging (MRI) was performed, which showed no abnormalities. Considering the parturient’s sensitivity to the trauma of the epidural puncture, a recommendation was made for heat application and continued observation. On the third day after surgery, the patient’s headache worsened, and the obstetrician considered that a postdural puncture headache (PDPH) had occurred. The obstetrician, drawing on his clinical experience, advised the patient to take ibuprofen orally for pain relief, to lie flat without a pillow to reduce further leakage of CSF, and to undergo intravenous fluid therapy. On the fourth postoperative day, the parturient’s headache did not subside, and symptoms of blurred vision and decreased acuity appeared, with bedside blood pressure at 165/95 mm Hg. The obstetrician immediately requested a neurology consultation. The neurologist recommended performing a cranial susceptibility weighted imaging + magnetic resonance venography scan. Concurrently, a lumbar puncture was performed to measure pressure, and biochemical analysis of the CSF was conducted, along with blood tests for the autoimmune system. The MRI report indicated abnormal signals in the bilateral temporal, parietal, occipital, and cerebellar hemispheres, with enhanced perfusion in the corresponding areas and a plump pituitary gland (Fig. 1).

**Figure 2.**
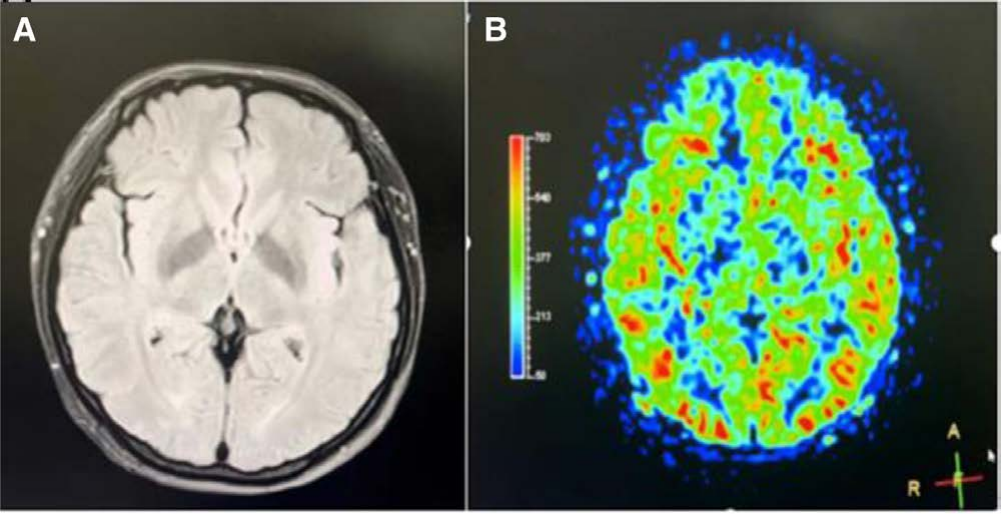
The cranial imaging findings of the patient following treatment and subsequent alleviation of clinical symptoms. (A) The abnormal signal intensity in the brain has resolved on MRI scan. (B) There are no significant abnormalities in cerebral blood perfusion on functional MRI. MRI = magnetic resonance imaging.

The CSF biochemical markers were within the normal range, and no abnormalities were found in the autoimmune antibodies. The neurologist considered the diagnosis of PRES. It was recommended to change the fluid therapy to restricted hydration, diuretics, blood pressure reduction, and neuronutrition. After active diuresis and blood pressure reduction, the parturient’s headache symptoms significantly improved, with blood pressure at 117/78 mm Hg, and vision gradually recovered. A week of treatment later, a follow-up cranial MRI showed that the lesions in the bilateral occipital lobes and cerebellar hemispheres had largely resolved (Fig. 2), further supporting the diagnosis of PRES.

## 3. Discussion

The subdural space is a potential gap situated between the dura mater and the arachnoid membrane, containing a small amount of serous fluid. It extends to the cranial cavity via the distribution of the meninges, covering all neural structures.^[5]^ Iatrogenic injury or congenital anatomical abnormalities may result in anesthesiologists inadvertently entering this space when inserting an epidural catheter or the medication spreading into this space, leading to specific clinical manifestations. Clinically, it is characterized mainly by widespread sensory block disproportionate to the local anesthetics used, delayed onset, complete or absent sensory block, and incomplete motor block.^[6]^ The current diagnostic methods rely primarily on clinical presentation and imaging examinations.^[7]^ Lubenow et al suggest that if 2 major criteria (negative CSF aspiration and extensive cephalic sensory block) and 1 minor criterion (delayed onset of more than 10 minutes and variable motor block or sympathetic block, Horner’s syndrome, or total spinal symptoms) are met, the probability of a subdural block is high.^[8]^ In reviewing our case, the patient received a small amount of medication but experienced a disproportionate, delayed high-level block, and the aspiration test for the catheter during anesthesia was negative. These findings meet the diagnostic criteria for a subdural block. As this was a retrospective analysis and the catheter was removed once the patient had left the operating room, further imaging evidence for diagnosis was not available in this case. This is also a limitation of this case.

PRES mainly presents with headaches, neurological deficits, seizures, or a range of visual impairments. The most common abnormalities on neuroimaging are vasogenic edema in the occipital white matter, sometimes extending to the adjacent parietal lobes.^[9]^ If PRES is detected early and the underlying cause is treated, most patients will make a recovery. In this case, the patient’s clinical symptoms and radiological findings are consistent with those of PRES. PRES is typically seen in cases of acute, severe hypertension or moderate but sudden blood pressure increases, or in individuals exposed to certain medications and toxins, especially among patients receiving chemotherapy and immunosuppressants. Both drug and toxic exposures may be linked to cerebrovascular dysregulation or endothelial dysfunction.^[10]^ In this instance, the patient had normal preoperative vital signs, no pregnancy-induced hypertension, no history of immunosuppressant use, normal preoperative liver and kidney function tests, and normal CSF biochemistry and blood autoimmune antibody levels at the onset. The only potential exposure factor considered is the intraoperative spread of local anesthetic towards the cranial side within the subdural space, causing reversible endothelial damage in the cerebral vasculature,leading to vasospasm, contraction, or abnormal autoregulation of blood perfusion, and resulting in postoperative vasogenic edema in the brain tissue. Some researchers have found that subdural block can lead to cranial spread disproportionate to the dose of local anesthetic, and there are reports of Horner’s syndrome and trigeminal nerve paralysis following such spread.^[11]^ Although the trigeminal ganglion is located intracranially, the mechanism of its blockade is not well understood, but it is clear that medication in the subdural space can lead to intracranial spread of local anesthetics, causing unpredictable changes in cranial nerve and vascular function.^[12]^

PDPH refers to the headache caused by a decrease in intracranial pressure due to the continuous leakage of CSF following the puncture of the dura mater during a lumbar puncture. The leakage of CSF reduces the support for brain tissue, leading to traction or compression of the structures that support the brain (such as the dura mater, blood vessels, and nerves), thereby causing headache and associated symptoms. There have been cases where PDPH has led to PRES, thought to be due to intracranial hypotension causing cerebral vasoconstriction.^[13–15]^ In this case, although PDPH was initially considered, the anesthetist confirmed that the 27-gauge spinal needle did not penetrate the dura mater during the procedure. Additionally, the fluid resuscitation administered by the obstetrician was ineffective, and postoperative imaging showed no evidence of CSF leakage. These factors essentially ruled out the possibility of PDPH causing PRES. Moreover, the patient’s unusual clinical manifestations during surgery suggested a subdural block, which was the most distinctive feature of this case. Therefore, the study still considers that an abnormal subdural block led to the abnormal high-level spread of local anesthetic, even into the cranial cavity, stimulating cerebral vasospasm and vasoconstriction, thereby triggering PRES. PRES usually has a favorable prognosis among pregnant women, with resolution being rapid and complete after adequate therapy Permanent damage can persist in a few cases (6%) and death due to hemorrhage has been described in a couple of patients.^[16]^ In this case, the obstetrician initially made an incorrect diagnosis of PDPH (post-dural puncture headache) and also administered the wrong treatment. Fortunately, they promptly recognized the worsening of the patient’s condition, and then made the correct diagnosis and treatment plan. The parturient had an excellent prognosis, with no neurological symptoms remaining, and was eventually discharged smoothly.

This case has enhanced our clinical experience, which can assist us in making diagnoses more swiftly and handling similar cases promptly based on those diagnoses in the future.

## Author contributions

**Conceptualization:** Jun He.

**Data curation:** Jun He.

**Project administration:** JianKe Kuai.

**Supervision:** JianKe Kuai.

**Writing – review & editing:** Jun He.

**Writing – original draft:** Haojie He.
